# MAEMC-NET: a hybrid self-supervised learning method for predicting the malignancy of solitary pulmonary nodules from CT images

**DOI:** 10.3389/fmed.2025.1507258

**Published:** 2025-02-12

**Authors:** Tianhu Zhao, Yong Yue, Hang Sun, Jingxu Li, Yanhua Wen, Yudong Yao, Wei Qian, Yubao Guan, Shouliang Qi

**Affiliations:** ^1^College of Medicine and Biological Information Engineering, Northeastern University, Shenyang, China; ^2^Key Laboratory of Intelligent Computing in Medical Image, Ministry of Education, Northeastern University, Shenyang, China; ^3^Department of Radiology, Shengjing Hospital of China Medical University, Shenyang, China; ^4^School of Information Science and Engineering, Shenyang Ligong University, Shenyang, China; ^5^Department of Radiology, The First Affiliated Hospital of Guangzhou Medical University, Guangzhou, China; ^6^Department of Radiology, The Fifth Affiliated Hospital of Guangzhou Medical University, Guangzhou, China; ^7^Department of Electrical and Computer Engineering, Stevens Institute of Technology, Hoboken, NJ, United States

**Keywords:** lung cancer, pulmonary granulomatous nodule, solid lung adenocarcinomas, CT image, self-supervised learning, masked autoencoder, momentum contrast

## Abstract

**Introduction:**

Pulmonary granulomatous nodules (PGN) often exhibit similar CT morphological features to solid lung adenocarcinomas (SLA), making preoperative differentiation challenging. This study aims to address this diagnostic challenge by developing a novel deep learning model.

**Methods:**

This study proposes MAEMC-NET, a model integrating generative (Masked AutoEncoder) and contrastive (Momentum Contrast) self-supervised learning to learn CT image representations of intra- and inter-solitary nodules. A generative self-supervised task of reconstructing masked axial CT patches containing lesions was designed to learn intra- and inter-slice image representations. Contrastive momentum is used to link the encoder in axial-CT-patch path with the momentum encoder in coronal-CT-patch path. A total of 494 patients from two centers were included.

**Results:**

MAEMC-NET achieved an area under curve (95% Confidence Interval) of 0.962 (0.934–0.973). These results not only significantly surpass the joint diagnosis by two experienced chest radiologists (77.3% accuracy) but also outperform the current state-of-the-art methods. The model performs best on medical images with a 50% mask ratio, showing a 1.4% increase in accuracy compared to the optimal 75% mask ratio on natural images.

**Discussion:**

The proposed MAEMC-NET effectively distinguishes between benign and malignant solitary pulmonary nodules and holds significant potential to assist radiologists in improving the diagnostic accuracy of PGN and SLA.

## 1 Introduction

Lung cancer is the leading cause of cancer-related deaths worldwide, responsible for more deaths than breast, prostate, and colon cancers combined (1). Each year, millions are diagnosed with lung cancer, with a significant portion identified at advanced stages where treatment options become limited and prognosis worsens (2). Early detection of lung cancer dramatically elevates survival rates and can mitigate the invasiveness and costs of treatments (3). Often, early-stage lung cancer presents with little to no discernible symptoms, meaning that by the time symptoms do emerge, the cancer may have metastasized (4). Given this, the pivotal role of routine screening, particularly via computed tomography (CT) scans known for their high resolution and sensitivity, becomes evident in enhancing early diagnosis (5).

Solitary pulmonary nodules (SPN) present as distinct, rounded opacities often smaller than 3 cm in diameter, typically encapsulated entirely within the pulmonary parenchyma without adjacent lung abnormalities (6). These radiographic features, while seemingly straightforward, hide a more complex underlying reality. In the clinical realm, a significant proportion of malignant peripheral SPNs are identified as solid lung adenocarcinomas (SLA) (7). However, a confounding factor in SPN evaluation emerges with pulmonary granulomatous nodules (PGN). These benign lesions can sometimes demonstrate spiculated or lobulated appearances on CT scans, closely mimicking the characteristics of their malignant counterparts, making differentiation between the two an intricate task (8). The striking radiological similarities between PGN and SLA have led to a reliance on invasive diagnostic tools, primarily percutaneous needle biopsies, to obtain a definitive diagnosis (9). While effective, these procedures come with their own set of challenges, often escalating the discomfort, anxiety, and potential complications for patients. In this context, the potential of a purely CT imaging-based diagnostic method not only represents a step forward in terms of patient comfort but also holds the promise of expediting diagnosis and subsequent treatments, thus revolutionizing the clinical approach to SPN (3).

Computer-Aided Diagnosis (CAD), especially when bolstered by deep learning techniques, offers a novel approach to diagnosing lung conditions, such as differentiating isolated granulomas from adenocarcinomas. CAD’s consistent and objective evaluations often exceed the capabilities of human expertise, which can be affected by factors such as fatigue, biases, and limited data processing abilities (10). Notably, deep learning, a sophisticated branch of machine learning, swiftly scans countless image slices, identifying minor abnormalities with remarkable accuracy (11). Trained on extensive datasets, these algorithms differentiate benign and malignant SPNs, thereby minimizing unnecessary biopsies and enhancing diagnostic reliability (12). Lakhani et al. (13) developed a convolutional neural network (CNN) that could effectively classify pulmonary tuberculosis from chest radiographs. Lee et al. (14) innovated a deep learning-based CAD system that excels at localizing and diagnosing metastatic brain tumors thus redefining the efficiency of tumor identification. Rajkomar et al. (15) employed machine learning to predict patient outcomes, such as unexpected readmissions, using routinely collected data during hospital admissions. However, classifying pulmonary nodules is challenging as benign and malignant lesions share similar imaging features, like morphology, edge characteristics, and tissue density, especially in CT images. This complicates automated diagnosis compared to conditions like tuberculosis or brain tumors. A key limitation of using deep learning for this task is the need for large, accurately annotated medical datasets, which require significant time and financial resources to obtain (16).

In recent years, the advent of self-supervised learning (SSL) has been recognized as a potential solution to the challenges posed by the need for meticulously annotated medical data (17). Distinguished from traditional supervised learning, SSL capitalizes on unlabeled data, deriving proxy tasks from the data itself to train models without human annotation (18). This technique not only alleviates the constraints and costs associated with data labeling but also harnesses the vast volumes of unlabeled medical images available, often yielding results comparable to, if not surpassing, supervised methods (19). In the realm of SSL, two dominant paradigms have notably emerged: generative and contrastive SSL.

**Figure 1:**
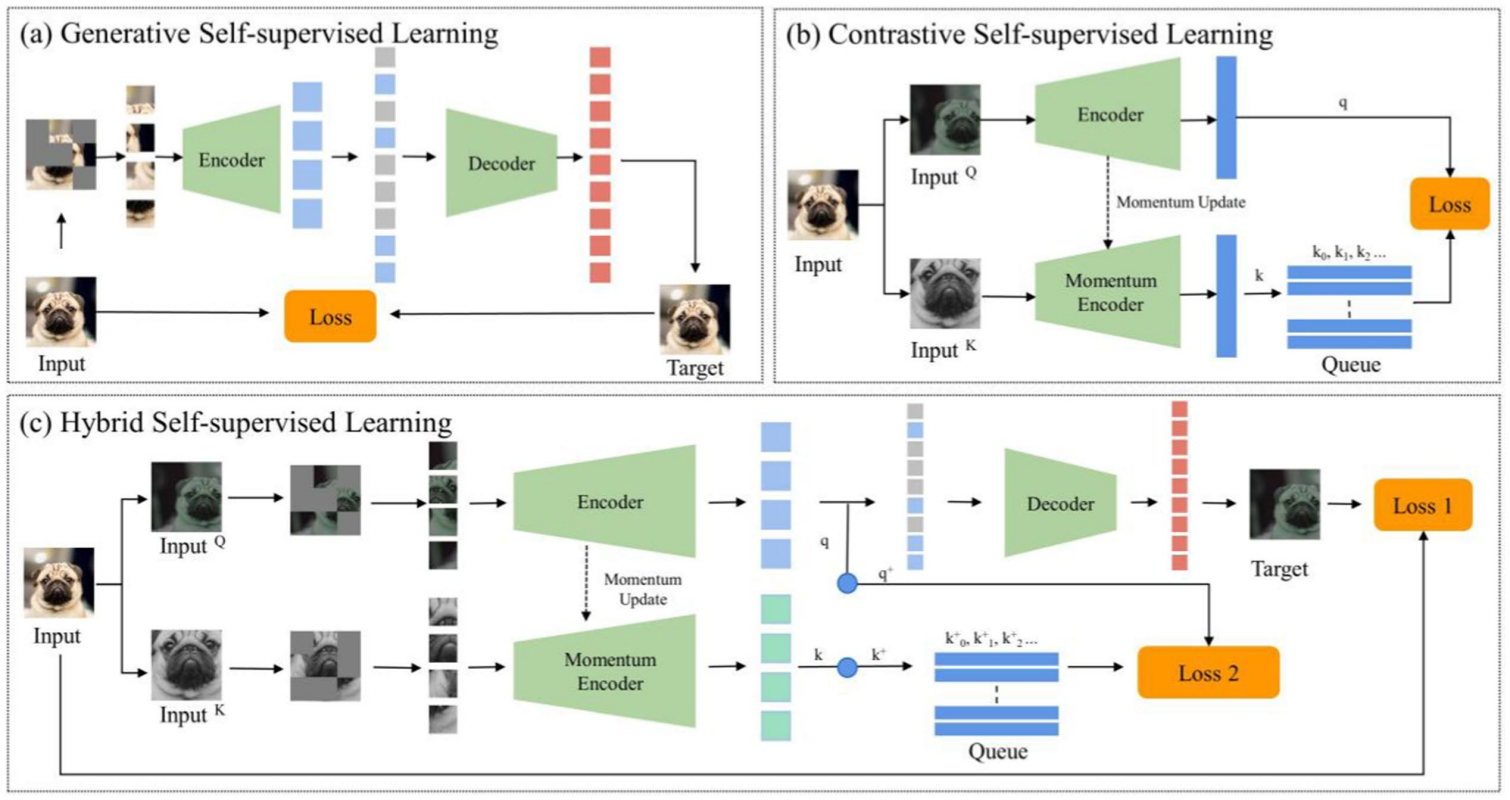
The general structure of self-supervised learning. **(A)** Generative self-supervised learning; **(B)** Contrastive self-supervised learning; **(C)** The proposed hybrid self-supervised learning.

Despite the pivotal advancements in self-supervised learning, two significant challenges persist. These challenges are often overlooked in prevailing literature: The first challenge pertains to the disparities between natural and medical imaging modalities. Medical images predominantly stem from radiographic, functional, magnetic resonance, and ultrasonic imaging modalities, whereas natural images are primarily captured through ambient light. This distinction underscores significant disparities, affecting both the application and the design of algorithms. Medical images are typically acquired through controlled environments with specific imaging devices, resulting in highly standardized formats, such as 3D single-channel grayscale representations for CT scans. In contrast, natural images come from natural environments and capture a broad spectrum of colors and structures under varying lighting conditions. This divergence impacts the algorithms designed for image classification. For example, traditional deep learning models are optimized for 2D, color-rich natural images, making it difficult to apply the same models to 3D grayscale medical images without losing crucial information. Additionally, natural images often contain a wide range of textures and diverse features that are not typically observed in medical images, where anatomical structures and subtle pathological variations are paramount. Additionally, the high similarity among medical images from the same anatomical region, even in healthy subjects, presents another unique challenge: minute differences between benign and malignant nodules may appear nearly identical, requiring algorithms to identify subtle variations that are critical for accurate diagnosis. The challenge here is evident: leveraging the success of SSL techniques in natural imaging for medical applications requires a comprehensive re-evaluation from a radiological perspective, followed by the formulation of a tailored approach. The second challenge involves the dichotomy between generative and contrastive SSL methodologies. Generative SSL focuses on pixel reconstruction, calculating the loss between the generated and original image, emphasizing intra-instance feature variations (Figure 1A). Conversely, contrastive SSL focuses on constructing positive and negative sample pairs, thereby determining the contrastive similarity metric and emphasizing inter-instance feature differences (Figure 1B), Given the inherent complexity and diverse feature information within medical images, integrating these two SSL methodologies could prove beneficial.

Given the distinct challenges arising from the disparities between natural and medical images and the nuanced differences between contrastive and generative SSLs, conventional neural networks often struggle in classifying pulmonary granulomatous nodules and solid lung adenocarcinomas. Consequently, developing a specialized self-supervised learning model that is custom-designed for this task and adept at navigating these challenges is crucial. To this end, we propose MAEMC-NET, an innovative self-supervised learning network conceived to address the limitations of existing methodologies. Figure 1C shows an approximate structure of the model. MAEMC-NET is designed for medical image classification, particularly focusing on differentiating Pulmonary granulomatous nodules and solid lung adenocarcinomas from CT scans of lung cancer patients. The architecture of MAEMC-NET integrates several critical components to achieve its outstanding effectiveness. Firstly, and most importantly, we have curated sample pairs by extracting regions of interest (ROI) from the axial and coronal planes of each patient case, thus ensuring a comprehensive and diverse dataset. Subsequently, the MAEMC-NET model, a hybrid of SSL strategies, effectively combines the strengths of both generative and contrastive SSLs. This integration allows the MAEMC-NET to exploit the capability to distinguish between inter-instance feature differences while also emphasizing intra-instance feature variations. Additionally, an optimized transformer module encoder has been developed, incorporating a dimensionality reduction module. This design enables a more effective synergy of contrastive and generative SSL techniques, further enhancing the performance of the model.

In this study, we present several key contributions: (1) We integrate generative and contrastive self-supervised learning techniques to develop robust CT image representations for both intra-and inter-solitary pulmonary nodules. (2) A novel generative self-supervised task is introduced, focusing on reconstructing masked axial CT patches that encompass lesions. This approach enhances our understanding of intra-inter-slice image representation. (3) We employ contrastive momentum to establish a connection between the encoder used in the axial-CT-patch pathway and the momentum encoder in the coronal-CT-patch pathway, thereby improving the coherence of our model. (4) To facilitate the computation of infoNCE loss in contrastive learning, we introduce a feature flattening module. This addition streamlines the processing and enhances the effectiveness of our methodology. (5) Through extensive comparative and ablation studies, we demonstrate the superior performance of our proposed model in predicting the malignancy of solitary pulmonary nodules.

## 2 Related works

### 2.1 Generative SSL and its applications in the medical field

Generative SSL has emerged as a powerful paradigm in machine learning, particularly in the medical domain, where labeled data is often scarce and expensive to obtain. This approach revolves around training models to understand the underlying data distribution by generating or reconstructing data samples without explicit supervision. In the medical field, where accurate diagnosis and interpretation of images are paramount, generative self-supervised learning techniques play a crucial role in tasks such as disease classification, anomaly detection, and image reconstruction. Figure 1A shows an approximate structure of the model.

One of the seminal contributions in generative SSL is the Variational Autoencoder, proposed by Kingma et al. (20), which introduced a mechanism to approximate complex data distributions using a probabilistic framework. Meanwhile, Creswell et al. (21) ushered in a transformative approach with Generative Adversarial Networks, wherein a duo of neural networks (generator and discriminator) engage in an adversarial game to create synthetic data that closely mirrors real data. The Denoising Autoencoder, conceptualized by Vincent et al. (22), is designed to reconstruct slightly corrupted input data. By prioritizing the reconstruction of this perturbed data, the model naturally learns to capture essential structures while discarding noise. Another significant contribution is a masked autoencoder architecture for scalable learning in computer vision tasks (23). This approach emphasizes the utilization of sparse representations and convolutional layers to facilitate efficient feature extraction and dimensionality reduction in large-scale visual datasets.

In the medical domain, generative SSL has been instrumental in various applications. A notable example is the work of Chen et al. (24), which introduces a SSL method for medical image analysis through image context restoration. Their model is trained to restore missing or corrupted regions in images, thereby enhancing representation learning for medical imagery. Taleb et al. (25) have also contributed to this field with their SSL framework based on image reconstruction. Using a generative model, their approach learns representations by reconstructing medical images, proving effective across various medical imaging tasks. Hu et al.’s research introduces Differentiable Architecture Search (DARTS) for 3D medical image analysis (26). Although not strictly a generative SSL method, it employs SSL to optimize neural network architectures, thereby enhancing performance in medical image tasks. Zhu et al. (27) have developed DeepEM, a method for weakly supervised pulmonary nodule detection using SSL. The model utilizes self-generated pseudo-labels and reconstructs 3D image patches, leading to improved nodule detection in medical imaging.

In a different application, Halimi et al.’s work, although not in the medical sector, introduces a self-supervised approach for dense correspondence learning (28). This method is significant for medical image-based 3D reconstruction tasks, enabling the generation of 3D models from 2D medical images. Another study by Chen et al. (29) explores a generative SSL approach where a network is trained to predict pixel values in medical images. This enhances the network’s capability to comprehend complex image structures. Lastly, Taleb et al.’s research focuses on the use of 3D medical images for SSL (30). Their model learns to reconstruct 3D medical scans, aiding in tasks like anomaly detection.

In summary, generative SSL is rapidly transforming the landscape of medical image analysis. Through innovative approaches like image context restoration, image reconstruction, optimization of neural network architectures, and 3D image processing, researchers are pushing the boundaries of what’s possible in medical diagnostics and treatment planning. These advancements not only demonstrate the versatility and power of generative SSL in handling complex, unlabeled medical data, but they also pave the way for more accurate, efficient, and accessible healthcare solutions. Despite promising results, generative SSL faces limitations in medical imaging. Models like variational autoencoders and GANs struggle with capturing fine-grained pathological features and handling the complexities of medical images. Moreover, unsupervised learning may lead to suboptimal representations when data distributions differ from the training data, highlighting the need for more specialized models.

### 2.2 Contrastive SSL and its applications in the medical field

Contrastive SSL is a potent technique in machine learning, particularly within computer vision. It operates by discerning positive and negative pairs of data samples, optimizing neural architectures to minimize the distance between positive pairs while maximizing that between negative pairs. Figure 1B shows an approximate structure of the model. Several influential frameworks have emerged within this approach: SimCLR, pioneered by Chen et al. (31), harnesses data augmentations to extract positive pairs from identical images, demonstrating robust performance across various visual representation tasks. Meanwhile, the Momentum Contrast method, introduced by He et al., extends SimCLR by utilizing a momentum-based encoder to dynamically update a sample dictionary during contrastive learning, particularly effective in handling restricted dictionary sizes (19). SimCLR and Momentum Contrast work well with 2D images but face challenges with 3D medical images. For lung CT images, 3D-specific augmentations like rotation, translation, and scaling are needed to maintain spatial consistency and capture texture details. Additionally, Grill et al. (32) innovatively introduced Bootstrap Your Own Latent (BYOL), which eliminates the need for negative pairs altogether. Instead, it utilizes two differently augmented views of the same image, simplifying the learning process while maintaining comparable performance.

In the medical field, contrastive SSL has made significant contributions, particularly in disease classification, interpretation, and various medical imaging tasks. For instance, Chen et al. (24) proposed a new self-supervised learning strategy, a context-based recovery strategy, which can effectively learn the semantic features of medical images without labeled data and significantly improve the performance of the model in tasks such as classification, localization, and segmentation. In another approach, Zhang et al. (33) The ConVIRT model is proposed, which is an unsupervised learning method that combines medical images and natural language description text. It can learn effective visual representations of medical images and performs well in multiple medical image tasks. In addition, compared with the traditional ImageNet pre-trained model, the ConVIRT method not only performs better in classification tasks, but also is more efficient in data utilization, especially when labeled data is scarce. Similarly, Zhuang et al. (34) adopted an innovative strategy by training a model on 3D medical images to solve a Rubik’s cube-like puzzle, facilitating the extraction of rich, transferable features for pathology analysis. Moreover, advancements in contrastive SSL have led to novel applications in histopathology image analysis. Srinidhi et al. (35) introduced an innovative framework that integrates task-agnostic self-supervised pre-training with task-specific semi-supervised learning consistency strategies. This approach has led to substantial advancements in image analysis tasks within computational pathology, particularly in situations where labeled data is limited. In cardiology, The CLOCS method proposed by Kiyasseh et al. (36) is an improved contrastive learning approach that effectively utilizes unlabeled physiological data. By performing contrastive learning across space, time, and patients, it learns more robust data representations. This method demonstrates excellent performance in various downstream tasks, especially when labeled data is scarce, offering strong generalization capabilities and accurate patient-specific predictions, with broad potential for application. Additionally, Xie et al.’s work on SSL of graph neural networks, and Wang et al.’s research in molecular contrastive learning of representations via graph neural networks have opened new avenues in molecular biology and chemistry (37, 38).

These diverse applications of contrastive SSL in the medical field not only highlight its versatility but also underscore its potential to revolutionize medical imaging and diagnostics. The ability to leverage unlabeled data effectively, understand complex image-text relationships, and extract meaningful features from various medical data points to promising advancements in diagnostic accuracy and efficiency. Despite its potential, contrastive SSL faces challenges in handling complex medical data and class imbalances, leading to suboptimal feature representations. Additionally, it requires large negative samples, which are often scarce in medical datasets. These issues suggest that integrating both generative and contrastive SSL methods could improve model performance.

## 3 Materials and methods

### 3.1 Dataset collection and characteristics

In order to comprehensively investigate the classification process of PGN and SLA, our research team collaborated with the First Affiliated Hospital of Guangzhou Medical University and Shengjing Hospital of China Medical University. We conducted a retrospective collection of data from 494 cases.

#### 3.1.1 Inclusion and exclusion criteria

The inclusion criteria for cases were as follows: (1) Patients with confirmed pathological diagnoses of pulmonary granulomatous lesions (tuberculous or fungal granulomas) and adenocarcinomas through surgical resection or image-guided biopsies. (2) All patients underwent routine and contrast-enhanced CT scans of the entire chest using the same CT machine and standardized reconstruction parameters within 2 weeks post-surgery. (3) Isolated solid SPNs with sizes ranging from 7 to 30 mm, without calcification or fat content, exhibiting characteristics such as spiculation, lobulation, or pleural indentation, and without associated lung atelectasis or lymph node enlargement. (4) Preoperative laboratory analysis of routine tumor markers (CEA, CA125, CA153) within 1 week before surgery, with positive thresholds set at >5 ng/mL, >35 ng/mL, and > 25 ng/mL, respectively, according to our institution’s reference ranges.

**Figure 2:**
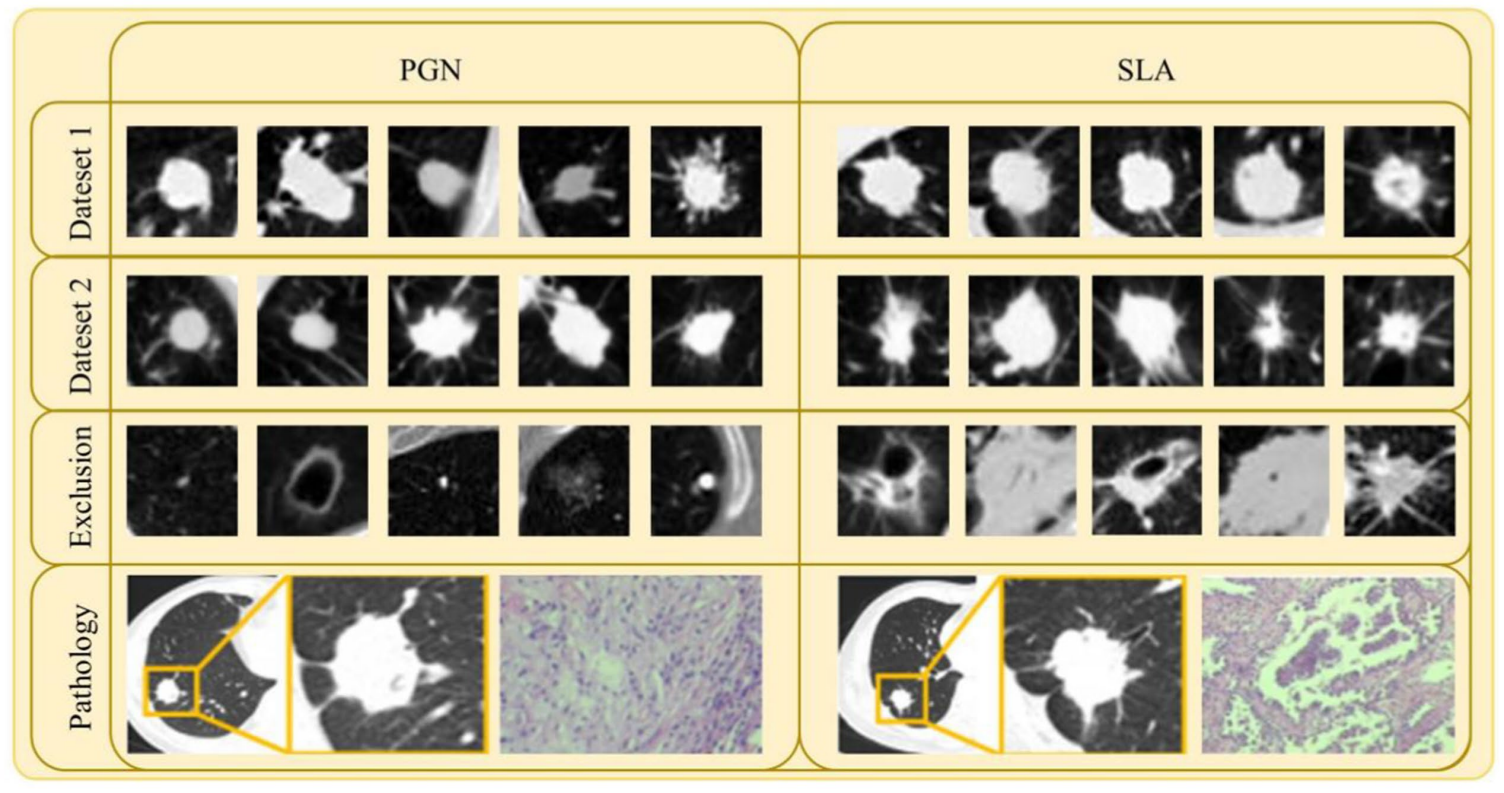
Samples of lesion images (CT and pathology) and excluded lesion images for PGN and SLA in Dataset 1 and Dataset 2.

Exclusion criteria included: (1) Nodules with features highly suggestive of benign lesions, such as caseous necrosis with cavitation in tuberculomas or characteristic halo sign in fungal granulomas. These features are typically associated with granulomatous inflammation or infection, which are non-malignant in nature, and could lead to misclassification of malignant lesions if included. (2) Individuals with a history of other malignant tumors or concurrent malignant tumors. (3) Cases imaged using different algorithms, different slice thicknesses, or on different CT machines. Some typical examples (both CT and pathology images) can be seen in Figure 2.

#### 3.1.2 Data collection and preparation

Among these cases, 333 cases were sourced from the First Affiliated Hospital of Guangzhou Medical University, comprising 105 cases of PGN and 228 cases of SLA. This specific dataset, designated as Dataset 1, served as the basis for model training, testing, and validation. Additionally, an additional 161 cases were gathered from Shengjing Hospital of China Medical University, encompassing 67 cases of PGN and 94 cases of SLA. This external dataset, referred to as Dataset 2, was utilized for external model validation to assess the generalization capability of the model. All CT images of the patients were acquired using a multi-detector CT system (AS+ 128-Slice; Siemens Healthineers, Germany). For Dataset 1, the CT scan parameters were set as follows: Tube Voltage at 120 kVp, Tube Current at an average of 299.88 mAs with a standard deviation of ±134.62 mAs, and an average Slice Thickness of 2.31 mm with a standard deviation of ±0.71 mm. In the case of Dataset 2, the parameters included a Tube Voltage of 120 kVp, a Tube Current averaging 194.31 mAs with a standard deviation of ±116.13 mAs, and an average slice thickness of 4.03 mm with a standard deviation of ±1.57 mm. All images from these datasets were exported in the DICOM format and subsequently utilized for image feature extraction by the MAEMC-NET model.

Two experienced chest radiologists, each possessing 10 and 20 years of experience in interpreting chest images, independently reviewed all CT images stored within our Picture Archiving and Communication System. Any discrepancies were resolved through discussion and consensus. Lesion Size was defined as the maximum diameter of the tumor in the axial image. Spiculation is defined as the presence of linear or pointed extensions that emanate from the edge of a nodule or mass and extend into the lung parenchyma, without reaching the pleural surface. Lobulation is characterized by a wavy, lobulated structure on a portion of the surface of the lesion, excluding areas adjacent to the pleura. Pleural Indentation is identified as a linear structure that originates from the tumor and extends to the pleural surface. These morphological assessments offer essential information for the subsequent analysis and classification of PGN and SLA. We have summarized the clinical characteristics of Dataset 1 in Table 1.

**Table 1:** Clinic characteristics of dataset 1.

Clinic characteristic	PGN (*n* = 105)	SLA (*n* = 228)	*p* value
Age, mean ± SD, years	50.02 ± 12.05	58.66 ± 12.63	<0.001*^a^
Age, <50/≥50, years	49/56	50/178	<0.001*^b^
Gender, Male/Female	68/37	118/110	0.009*^b^
Spiculated sign, Yes/No	72/33	155/73	0.930^b^
Lobulated sign, Yes/No	63/42	177/51	0.001*^b^
Pleural retraction sign, Yes/No	28/77	139/89	<0.001*^b^
Lesion size, mean ± SD, cm	1.81 ± 0.62	2.05 ± 0.34	<0.001*^a^
kVp, kV	120	120	–
Slice thickness, mm	2.25 ± 1.32	2.34 ± 1.07	<0.001*^a^
X-ray tube current, mean ± SD, mA	311.28 ± 159.76	294.63 ± 114.27	<0.001*^a^

*Indicates that the significance is available. ^a,b^ indicates the two-sample *t*-test and Chi-square test, respectively.

This retrospective research received approval from our institution’s Institutional Review Board, and all procedures were conducted in accordance with the ethical guidelines established by the institution.

### 3.2 Data processing

To assure the quality and uniformity of the dataset for training and evaluation purposes, a comprehensive series of data processing and augmentation techniques was employed. The preprocessing of the CT images involved critical steps such as standardizing resolution, reducing noise, and enhancing contrast. These measures were instrumental in ensuring dataset consistency and improving the quality of the input data, which is crucial for the subsequent stages of analysis.

First, given the variability in slice thickness across different scans, an initial step involved the resampling of all CT images to a uniform slice thickness of 1 mm using trilinear interpolation. This meticulous process guaranteed that all images conformed to a consistent format, facilitating seamless subsequent analysis. Furthermore, a critical aspect of the preprocessing pipeline was intensity normalization. The CT images underwent intensity normalization to alleviate variations in image intensity attributed to differences in acquisition settings. This calibration involved standardizing the Hounsfield units (HU) to establish a uniform intensity scale across all images. As a result, all CT data were uniformly configured with a window width of 1,400 HU and a window level of −500 HU. Additionally, to reduce interference from surrounding information and focus specifically on the lesions, we selected the center point of each lesion as an anchor. We then extracted square ROI regions with a side length of 50 mm to ensure the entire tumor region, including surrounding tissue or irregularities, was covered. This size accommodated variations in tumor size, capturing both small and large lesions, and prevented clipping of the tumor, ensuring complete coverage. A total of 25 slices containing the tumor lesion were obtained from both the axial and coronal planes. Subsequently, these slices from the axial and coronal planes were sequentially arranged to generate two sets of 5 × 5 sample pairs, which were used as the training dataset for the model.

To mitigate the risk of overfitting during model training, a variety of data augmentation techniques were applied to enlarge the dataset. This approach successfully enhanced the diversity of the dataset, thereby improving the model’s ability to generalize. In this study, specific augmentation techniques such as rotation, horizontal flipping, and four-directional translations centered on the central anchor point were implemented, resulting in the generation of additional valuable data. The incorporation of these techniques substantially increased the dataset size, leading to a total of 13,320 sample pairs in Dataset 1. Subsequently, Dataset 1 was utilized for the self-supervised pre-training of the MAEMC-NET model. During the fine-tuning phase in downstream tasks, the unexpanded original dataset was employed for training, where the data was split into training and testing sets in an 8:2 ratio and subjected to five-fold cross-validation. Dataset 2 was utilized as an external validation set to independently assess the performance of the model.

### 3.3 Architecture of MAEMC-NET

**Figure 3:**
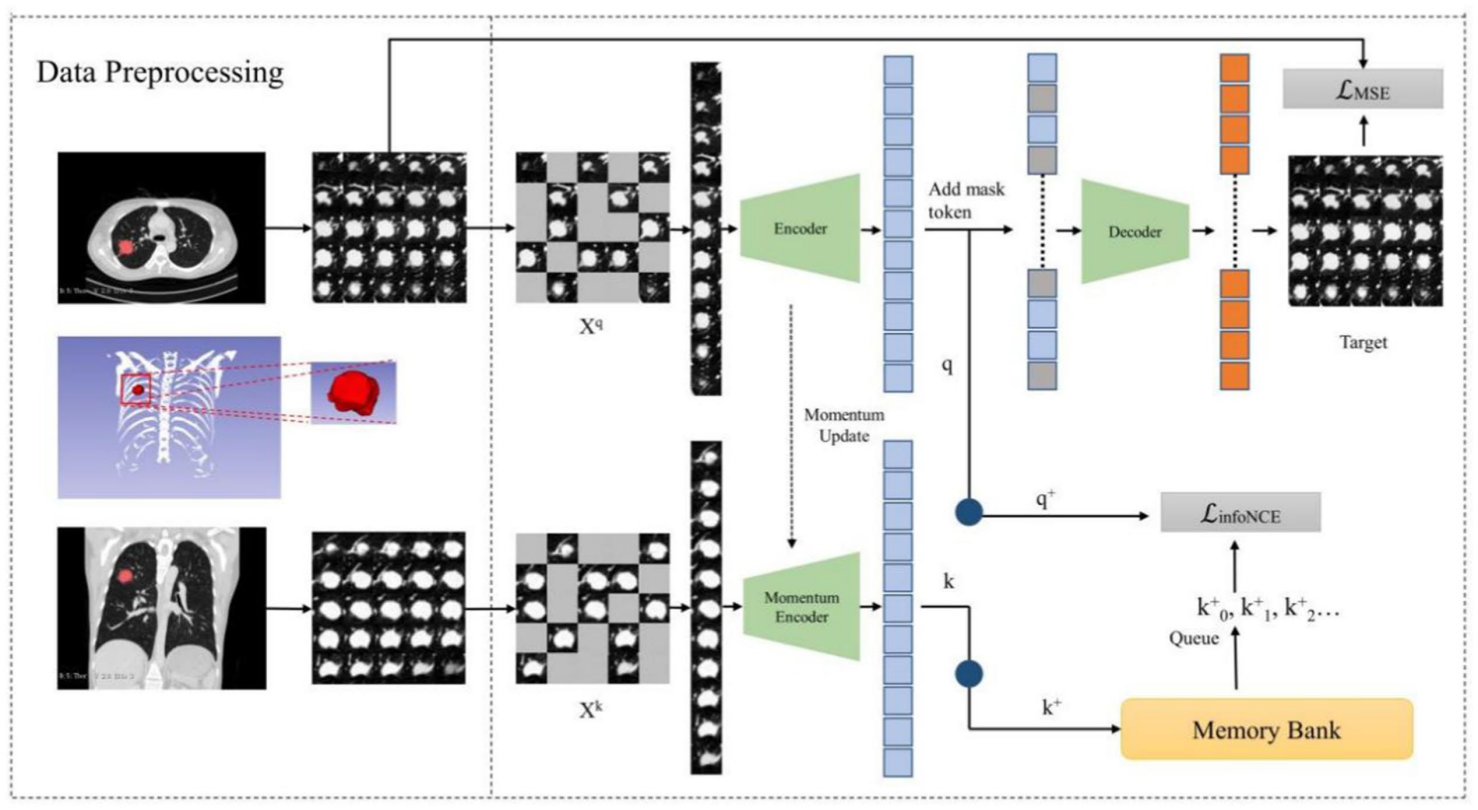
The overview of our proposed MAEMC network.

In our study, we introduce the MAEMC-NET, a novel self-supervised learning network tailored for CT image-based classification of PGN and SLA. This innovative network uniquely combines generative and contrastive self-supervised learning. A custom-designed pretext task is also developed, ensuring a perfect fit for the classification requirements. Figure 3 in our paper provides a detailed overview of the methodology, comprising three integral components: a data processing module, a generative self-supervised learning task module, a contrastive self-supervised learning task module. These modules collaboratively work to significantly enhance the accuracy and efficiency of PGN and SLA classification in CT imaging.

#### 3.3.1 Data processing module and position embedding

In alignment with the Vision Transformer (ViT) architecture and the intermediate task designed for this experiment, we amalgamated training set images composed of multiple lesion images (39). They were derived from a previously obtained preprocessed dataset extracting sample pairs from multi-slice axial and coronal views of the lesion. We divided these composite images into regular, non-overlapping patches, treating each patch independently. Specifically, we partitioned the 250 × 250 images into 25 uniform, non-overlapping 50×50 patches, each encompassing a portion of the lesion image. Subsequently, these patches underwent uniform, randomly distributed sampling, with a certain proportion being masked.

Following this, we employed linear projection to obtain token embeddings for the unmasked patches. To preserve vital positional information, we incorporated positional embedding techniques. For this purpose, we adopted the Sinusoidal Position Embedding method detailed in reference [40]. This method involves adding sinusoidal waves of varying frequencies to the embeddings of input tokens. The goal is to achieve the superposition of multiple cosine waves when performing inner products between tokens. This superposition effectively encodes the relative positional information between tokens, representing the spatial relationships within the image. The formula for Sinusoidal Position Embedding is presented below (Equations 1–2):


(1) $$PE\left(pos,2i\right)=\sin\left(\frac{pos}{10000^{2i/d_{\text{model}}}}\right)$$

(2) $$PE\left(pos,2i+1\right)=\cos\left(\frac{pos}{10000^{2i/d_{\text{model}}}}\right)$$

where $PE\left(pos,2i\right)$ and $PE\left(pos,2i+1\right)$ represent the positional embedding values for even $\left(2i\right)$ and odd $\left(2i+1\right)$ dimensions, respectively. pos signifies the specific position in the sequence, while $d_{\mathrm{mod}el}$ refers to the embedding dimension of the model. The application of this technique enhances the token embeddings by incorporating positional data, thereby enabling a more detailed and thorough representation of spatial relationships in the image.

#### 3.3.2 Hybrid self-supervised learning

In the realm of SSL, we delve into the synergy of contrastive SSL and generative SSL. Contrastive SSL, as the name suggests, uncovers distinctive image features by contrasting positive and negative instances within a high-dimensional space. On the flip side, generative SSL harnesses the power of image reconstruction to grasp valuable image information. Our observation revealed an interesting facet: the masked autoencoder (MAE) tends to adopt a global perspective when considering an image, while the momentum contrast (MoCo) method leans toward scrutinizing unique image regions, strategically positioning positive and negative examples.

MoCo is a contrastive SSL model known for its effectiveness in various visual tasks. It employs a unique strategy involving a dynamic dictionary and momentum-based updates. This method allows MoCo to efficiently learn robust and distinct features by contrasting positive and negative data samples. MAE is a generative SSL model renowned for its impressive performance in a range of visual tasks. It utilizes an asymmetric encoder-decoder architecture. Its success is largely due to the use of the Vanilla Vision Transformer, adept at extracting global features from input data. This architecture enables MAE to effectively reconstruct missing parts of the input, thereby learning comprehensive representations.

To optimize the training of our model and to enhance the capabilities of the Transformer, we introduce Hybrid SSL as a pivotal intermediary task within the MAEMC-NET. This strategic addition empowers the transformer to explore both the idiosyncratic characteristics and holistic image context. Our approach commences with the initialization of two encoders, E and ME, furnished with identical weights. However, it is important to note that ME experiences momentum-based updates. Subsequently, a series of random masking operations are applied to augmented images $X^{q}$ and $X^{k}$, both originating from the same lesion and sourced from multi-layered axial and coronal planes. This masking operation leads to the creation of mask vectors q and k.

On one front, vectors q and k undergo dimension reduction through individual one-dimensional convolution layers, resulting in $q^{+}$ and $k^{+}$, respectively. Notably, $k^{+}$ finds its residence in a queue-like memory bank, which continually evolves as new training batches are introduced, seamlessly replacing the oldest ones. Consequently, we employ the InfoNCE loss function to gage the similarity between $q^{+}$ and $k^{+}$, acting as the cornerstone of our comparative task. The formula for the InfoNCE loss function is as follows (Equation 3):


(3) $$L_{\text{InfoNCE}}=-\log\frac{\exp\left(q^{+}\cdot k_{+}^{+}/\tau \right)}{\sum_{i=0}^{k}\exp\left(q^{+}\cdot k_{i}^{+}/\tau \right)}$$

where $q^{+}$ serves as the query key, while $k_{0}^{+}$, $k_{1}^{+}$, $k_{2}^{+}$ act as keys in the dictionary. Among these, $q^{+}$ and $k_{+}^{+}$ are treated as positive sample pairs, whereas the other keys function as negative sample pairs for $q^{+}$. Additionally, τ represents a temperature parameter that controls the concentration level of the distribution. By minimizing the infoNCE loss, the model is trained to effectively distinguish between positive and negative sample pairs, thereby enhancing its capability to learn discriminative features from the data.

The combination of q with the mask token constitutes the input to the decoder module, which is tasked with predicting the pixel values of the reconstructed mask patch. Subsequently, the evaluation utilizes the Mean Squared Error (MSE) loss function to quantify the discrepancy between the predicted and original pixel values. The formula for the MSE loss function is as follows (Equation 4):


(4) $$L_{MSE}=\frac{1}{N}\sum_{i=1}^{N}{\left(X_{q}^{i}-\overset{\wedge }{X_{q}^{i}}\right)}^{2}$$

where N represents the total number of missing pixels in the original image, $X_{q}^{i}$ denotes the true pixel value of the i-th pixel, and $\overset{\wedge }{X_{q}^{i}}$ signifies the predicted pixel value by the model. By minimizing the MSE loss, the model is trained to accurately reconstruct each pixel, thus facilitating the learning of the global features of the image.

Finally, we combine the InfoNCE loss function with the MSE loss function using a specific temperature coefficient λ, to serve as the loss function to optimize the model. The formula for the loss function is as follows (Equation 5):


(5) $$L=\lambda \times L_{MSE}+\left(1-\lambda \right)\times L_{\text{InfoNCE}}$$

#### 3.3.3 Detailing encoder, decoder, and momentum contrast components

**Figure 4:**
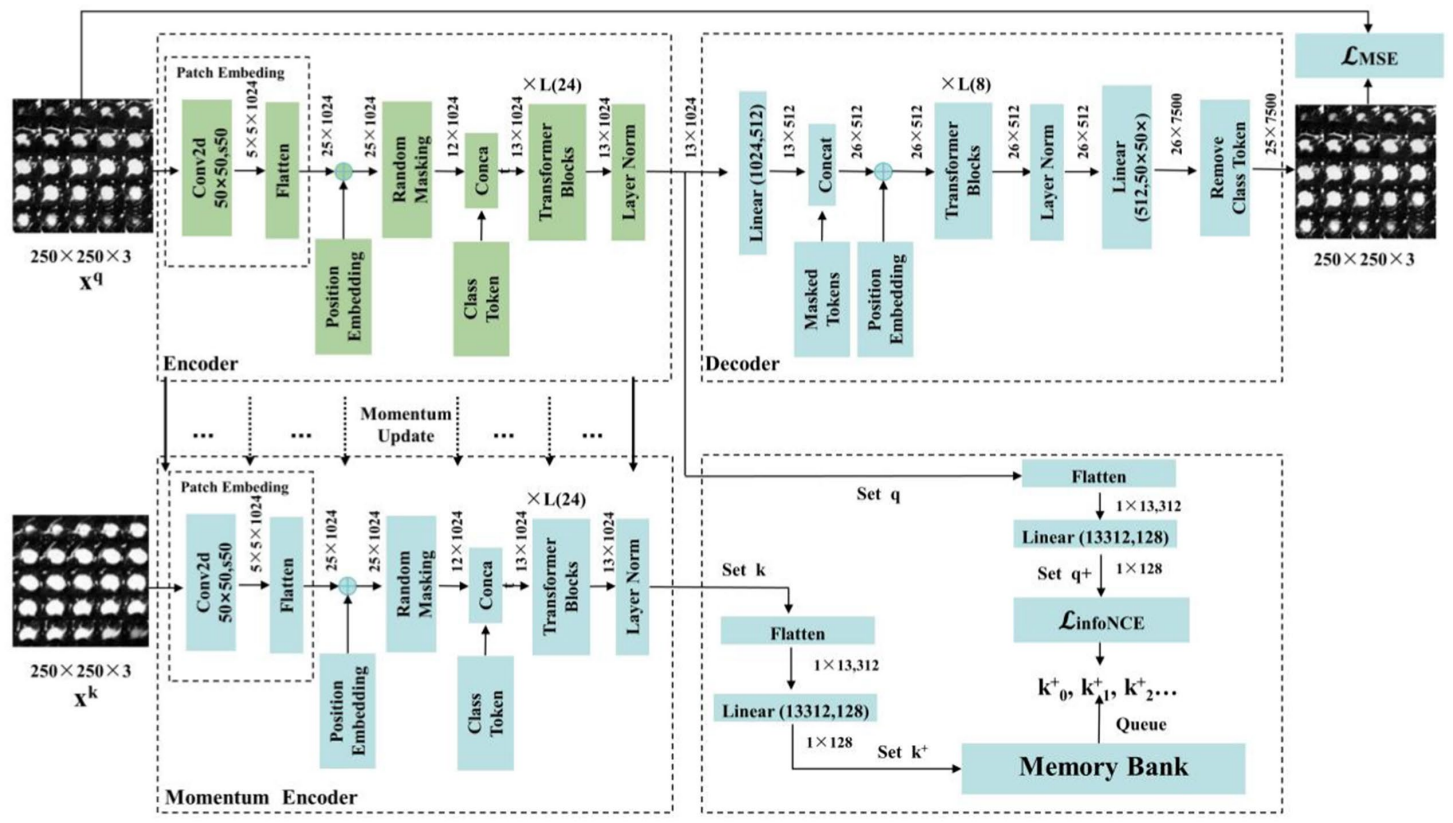
The structure of the MAEMC network and its details and parameters.

In this section, we introduce the components of the MAEMC-NET model, including the encoder module, decoder module, and momentum encoder module, along with the specifics of the momentum update operation and the implementation of the dictionary as a queue. The detailed network architecture of this module is visually represented in Figure 4. The encoder and momentum encoder modules utilize the ViT-large model, characterized by 24 stacked encoder blocks, a token vector length of 1,024, and 16 heads in the multi-head attention mechanism. These modules exclusively process un-masked patches. Initially, image data is segmented into patches of a specified size and then flattened into one-dimensional arrays. Subsequently, these arrays undergo a linear transformation, which is then merged with the position embeddings of the original image and augmented by the addition of a class token at the beginning. Owing to the asymmetric Encoder-Decoder architecture employed, where the encoder only processes un-masked patch information to conserve computational resources, the decoder module is configured with 8 stacked decoder blocks and a token vector length of 512. The decoder is tasked with processing not only the un-masked tokens encoded by the Encoder but also the masked tokens. It is important to note that these masked tokens are not derived from the embedded transformation of the previously masked patches; rather, they are learnable and shared across all masked patches.

In traditional contrastive learning, the size of the dictionary is typically equivalent to that of the mini-batch. However, this approach is often constrained by the limitations of GPU memory and computational power, preventing the use of large batch sizes. To address this limitation, the MoCo framework employs a queue-based mechanism to store the dictionary, which contains feature vectors of images, allowing for a substantially larger dictionary size. Nonetheless, in cases where the dataset size is not exceptionally large, selecting an overly extensive dictionary can impede model convergence. Therefore, in our implementation, the dictionary size is set to 700, with each vector having a dimensionality of 128. The maintenance of the queue involves enqueuing the feature vectors of the most recent batch of images and dequeuing those of the earliest batch.

To ensure consistency among the keys in the queue, it is imperative that the Momentum Encoder associated with the dictionary is updated gradually. This is achieved through the implementation of a momentum-based approach. Specifically, after each update of the encoder, only 1% of its updated parameters are used to modify the Momentum Encoder. Such a method ensures the slow and controlled update of the Momentum Encoder, maintaining the stability and consistency of the keys within the queue.

### 3.4 Performance evaluation measures

We selected commonly used evaluation metrics to assess the performance of our model. These include Area Under the Curve (AUC) (Equation 6) with 95% Confidence Interval (95% CI), Accuracy (ACC), Sensitivity (SEN), and Specificity (SPE). AUC quantifies the ability of the model to differentiate between classes.


(6) $$AUC=\int_{0}^{1}TPR\left(t\right)\text{dFPR}\left(t\right)$$

where TPR (True Positive Rate) and FPR (False Positive Rate) vary with different thresholds t. AUC is presented with its 95% CI, offering a statistical range indicating where the true AUC value is likely to lie with 95% confidence. This measure enhances the interpretability and reliability of the AUC metric.

ACC (Equation 7) reflects the overall effectiveness of the model in correctly classifying both positive and negative cases. SEN (Equation 8) plays a crucial role in determining the proficiency of the model in identifying true positive cases, which is essential for ensuring that no actual cases are overlooked. Conversely, SPE (Equation 9) assesses the capability of the model in accurately recognizing negative cases, a critical factor in minimizing the occurrence of false positives.


(7) $$ACC=\frac{TP+TN}{TP+TN+FP+FN}$$

(8) $$SEN=\frac{TP}{TP+FN}$$

(9) $$SPE=\frac{TN}{TN+FP}$$

where TP is True Positives, TN is True Negatives, FP is False Positives, and FN is False Negatives.

### 3.5 Training of the models and experiment setting

We incorporated the MAEMC-NET model, training and testing all variants and comparison models on an NVIDIA GeForce RTX 4070 with 12GB memory. This was implemented using PyTorch (version 1.7), with all graphics created using matplotlib in Python. Our network, an evolution of MAE and MoCo, pre-trained model parameters were not found to be loadable into MAEMC-NET, necessitating training from scratch. To mitigate overfitting, we implemented an early stopping mechanism during the fine-tuning phase. The validation loss was monitored after each epoch, and training was halted if no improvement was observed for 10 consecutive epochs. This helped in preserving the model’s generalization ability.

During pre-training stage, we used grid search to adjust hyperparameters such as learning rate, batch size, and optimizer settings. We tested different combinations of learning rates (1 × 10^−2^, 1 × 10^−3^, 1 × 10^−4^) and batch sizes (32, 64, 128) to determine the best configuration. Finally, the images size was 250 × 250 with a batch size set to 64. Optimization was carried out using the AdamW optimizer with betas = (0.9, 0.95), epochs set to 100 with an initial learning rate of 1 × 10^−2^, reduced by 0.1 at epochs 120 and 160. In the fine-tuning stage for downstream tasks, we utilized the pre-trained encoder module, adding a two-class fully connected layer, maintaining the same image size and batch size, with 100 epochs and an initial learning rate of 1 × 10^−3^, reduced by 0.1 at epochs 40 and 70.

## 4 Results

### 4.1 Performance of MAEMC-NET and counterparts

We evaluated the performance of our MAEMC-NET model, aimed at classifying PGN and SLA in CT images, against several state-of-the-art SSL methods, such as SimCLR (31), MoCo v1 (19), MoCo v2 (19), MAE (23), CMAE (41), and Convnext v2 (42). A supervised learning model using the ViT architecture served as the baseline for performance comparison.

**Figure 5:**
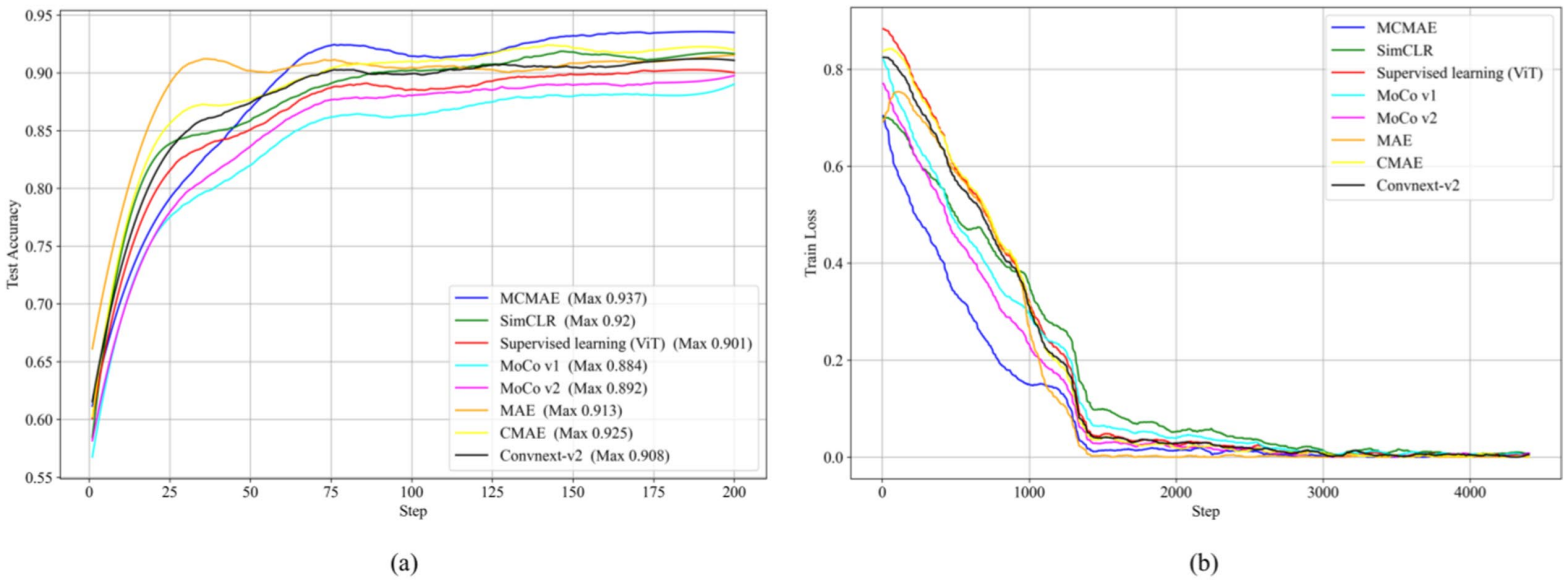
Comparison of the MAEMC-NET model and state-of-the-art SSL methods. **(A)** Test accuracy curves; **(B)** Training loss curves.

The test accuracy progression of each model over increasing epochs, as depicted in Figure 5A, highlights that our MAEMC-NET model, although initially moderate in performance, consistently achieved the highest test accuracy after epoch 75. Figure 5B presents the training loss curves of these models, indicating an initial slight increase in loss for the MAE model but eventual convergence to optimal accuracy for all models. Table 2 showcases the final results: MAEMC-NET achieved an AUC of 0.962 and an ACC of 93.7% on our PGN and SLA dataset, outperforming the baseline ViT model by 0.032 in AUC and 3.6% in ACC. Additionally, it improved upon the previously best-performing model, CMAE (AUC 0.957, ACC 92.5%), by 1.2% in ACC. It is noteworthy that in all models, SPE was higher than SEN, a reflection of the class imbalance in our dataset, which has a greater number of SLA compared to PGN. This imbalance is common in medical datasets, where malignant cases often outnumber benign ones. Despite this, our model also excelled in sensitivity and specificity, achieving the best results with 91.2 and 95.9%, respectively. To further evaluate the generalization capability of the MAEMC-NET model, we conducted an assessment using Dataset 2. The model achieved an AUC of 0.949 and an ACC of 91.5%. Additionally, the sensitivity and specificity were 90.3 and 92.8%, respectively. These results indicate that the MAEMC-NET model not only performs well on the original PGN and SLA dataset but also maintains high performance when applied to an independent external dataset, underscoring its robust generalization ability.

**Table 2:** Results of the comparison between MAEMC-NET and the state-of-the-art methods.

Method	AUC (95% CI)	ACC	SEN	SPE
Supervised learning (ViT) (39)	0.930 (0.912–0.947)	90.1%	87.3%	92.2%
MoCo v1 (19)	0.907 (0.883–0.929)	88.4%	85.8%	90.1%
MoCo v2 (19)	0.918 (0.894–0.942)	89.2%	86.5%	91.3%
MAE (23)	0.937 (0.915–0.959)	91.3%	88.7%	93.4%
SimCLR (31)	0.951 (0.927–0.975)	92.0%	89.4%	94.6%
CMAE (41)	0.957 (0.931–0.966)	92.5%	89.9%	94.3%
Convnext v2 (42)	0.935 (0.914–0.951)	90.8%	87.5%	92.6%
MAEMC-NET (Ours)	0.962 (0.934–0.973)	93.7%	91.2%	95.9%

### 4.2 Ablation analysis

Our MAEMC-NET model consists of three pivotal modules: Firstly, a data processing module (DP) designed for generating pretext tasks in pretraining. This module plays a crucial role in preparing the dataset for subsequent learning tasks. Secondly, the generative SSL (GSSL) module emphasizes pixel reconstruction with a global perspective, aiming to capture valuable image information efficiently. This approach proves to be essential in understanding the intricate details and broader context of medical images. Thirdly, the contrastive SSL (CSSL) module, which concentrates on discerning unique image features by contrasting high-dimensional instances, focusing especially on distinctive image regions. This method plays a pivotal role in enhancing the discriminative power of the model.

To assess the impact of each module on the classification of PGN and SLA in CT images, we conducted comprehensive ablation experiments. As indicated in Table 3, we initially replicated the MoCo model as a baseline for contrastive SSL and the MAE model for generative SSL. Both models were then enhanced using our data processing module for pretext tasks. This enhancement led to significant improvements in performance metrics, with notable increases in AUC and ACC for both models. We attribute this improvement primarily to our tailored pretext tasks and data processing techniques, which align more closely with the extraction of pathological features from medical images.

**Table 3:** Ablation studies for MAEMC-NET.

Method			AUC (95%CI)	ACC	SEN	SPE
DP	CSSL	GSSL				
	✓		0.875 (0.853–0.897)	86.7%	83.3%	89.6%
		✓	0.892 (0.870–0.914)	87.5%	84.7%	90.3%
✓	✓		0.907 (0.883–0.929)	88.4%	85.8%	90.1%
✓		✓	0.937 (0.915–0.959)	91.3%	88.7%	93.4%
	✓	✓	0.925 (0.903–0.947)	90.9%	87.8%	93.7%
✓	✓	✓	0.962 (0.934–0.973)	93.7%	91.2%	95.9%

Furthermore, we experimented with a hybrid model that combines both the MoCo and MAE models. This experiment yielded substantial improvements in performance over the individual models, validating the effectiveness of integrating contrastive and generative SSL approaches. The hybrid model capitalizes on the strengths of both learning strategies, merging the detailed recovery and contextual understanding characteristic of generative learning with the feature distinction and relational understanding central to contrastive learning.

Finally, our MAEMC-NET model, which incorporates all three modules, achieved the most superior results. This outcome further illustrates the indispensable role of each module in the overall performance of our model, highlighting the synergy achieved through their combination.

### 4.3 Optimizing mask ratios in MAEMC-NET

This section investigates the influence of various mask ratios on our MAEMC-NET model during its generative SSL phase. The mask ratio is an essential factor, representing the proportion of the input image that is masked or hidden during the training process. Our experiments focused on different ratios to strike a delicate balance between providing the model with a sufficient challenge for learning robust features, and retaining enough visible information to ensure effective reconstruction.

**Figure 6:**
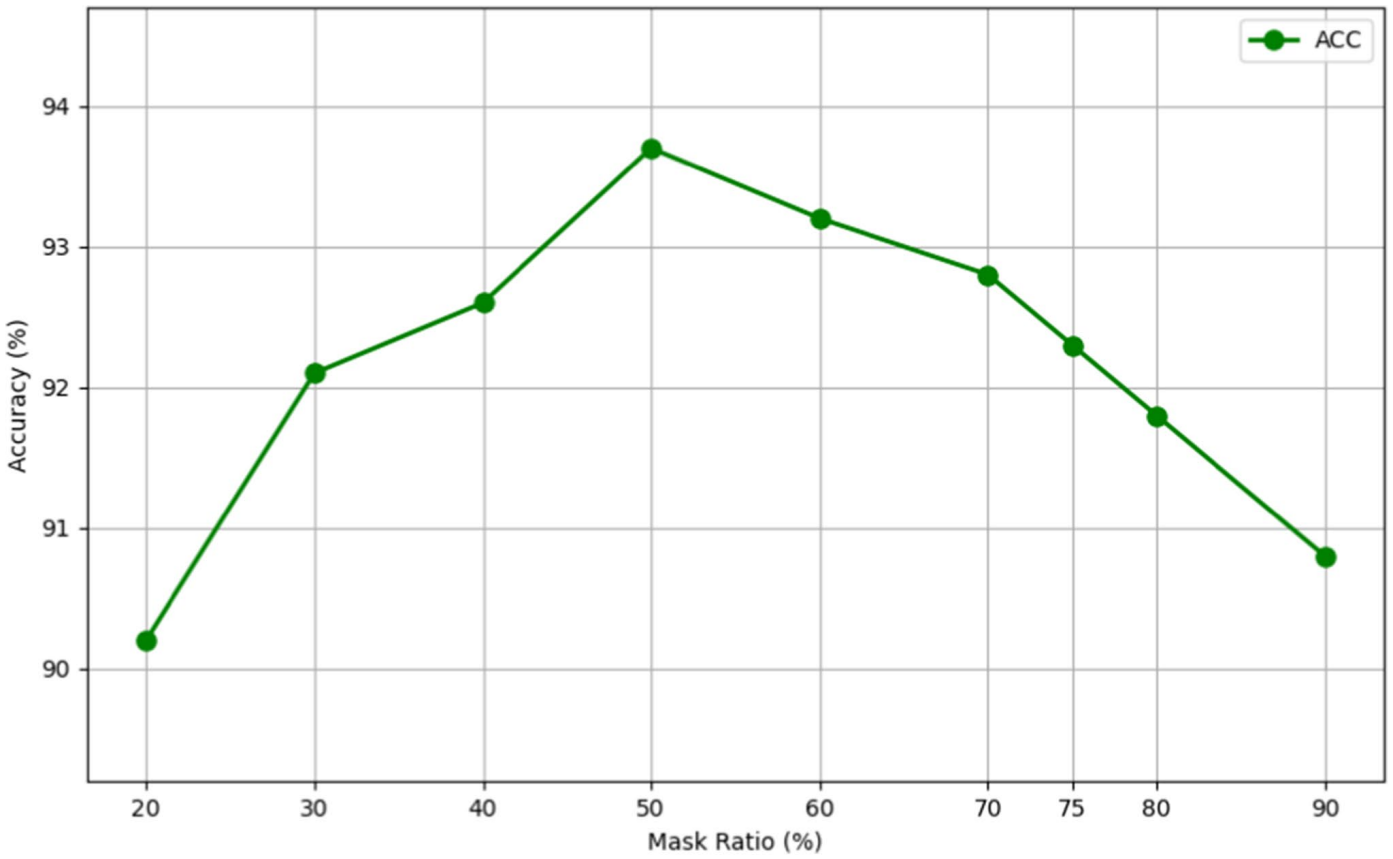
A line graph of the test accuracy of MAEMC-NET at different mask ratios.

As evidenced in Figure 6 and Table 4, we observed that a 50% mask ratio delivered the most favorable outcomes for our model on the CT image dataset, specifically in distinguishing between PGN and SLA. This finding stands in stark contrast to the optimal 75% mask ratio observed in applications involving natural images. We attribute this variance to the complex and dense nature of medical images. Lesion images, for instance, contain a wealth of tissue and pathological features, offering a higher information density compared to natural images. Therefore, the MAEMC-NET model requires access to more image information for the accurate reconstruction of medical images.

**Table 4:** The performance of MAEMC-NET under different mask ratios.

Mask Ratio	AUC (95%CI)	ACC	SEN	SPE
20%	0.933 (0.906–0.954)	90.2%	88.7%	91.8%
30%	0.941 (0.912–0.967)	92.1%	90.2%	93.5%
40%	0.954 (0.923–0.972)	92.6%	90.8%	93.4%
50% (Ours)	0.962 (0.934–0.973)	93.7%	91.2%	95.9%
60%	0.968 (0.935–0.973)	93.2%	91.6%	94.4%
70%	0.957 (0.924–0.961)	92.8%	90.3%	94.7%
75%	0.955 (0.923–0.965)	92.3%	89.8%	95.5%
80%	0.946 (0.910–0.957)	91.8%	89.3%	93.0%
90%	0.932 (0.907–0.948)	90.8%	88.4%	92.2%

Furthermore, our experimental results across different mask ratios have consistently shown superior performance of our model over other comparative models, regardless of the fluctuation in results due to varying mask ratios. This consistent outperformance underlines the robustness and adaptability of MAEMC-NET, further validating its superiority in handling medical image classification tasks.

### 4.4 Evaluation of different backbone networks in MAEMC-NET

The backbone network plays a pivotal role in our MAEMC-NET model, and this section provides an in-depth analysis of its impact on the performance of the model. We evaluated various networks, including Vit-base, Swin Transformer (43), DeiT (44), LeVit (45), PVT (46), and our customized Vit-Large, particularly focusing on their efficacy in classifying PGN and SLA in CT images. The experimental results are shown in Table 5, and the assessment utilized metrics such as AUC, ACC, SEN, and SPE.

**Table 5:** Comparison of classification performance with different backbones on the dataset 1.

Backbone	AUC (95%CI)	ACC	SEN	SPE
Vit-base (23)	0.927 (0.903–0.951)	90.3%	87.9%	92.7%
Swin Transformer (43)	0.943 (0.921–0.965)	91.9%	89.4%	94.3%
DeiT (44)	0.938 (0.915–0.961)	91.6%	89.1%	93.9%
LeVit (45)	0.947 (0.929–0.975)	92.3%	90.2%	95.1%
PVT (46)	0.943 (0.924–0.970)	92.1%	89.7%	94.6%
Vit-Large (Ours)	0.962 (0.934–0.973)	93.7%	91.2%	95.9%

Our modified Vit-Large backbone network stood out, showcasing an enhanced ability in processing the intricate details of medical imaging data. Unlike other networks such as Swin Transformer and DeiT, which have their strengths, they not uniformly extract features across resolutions or effectively capture the subtle nuances typical in medical images. In comparison to Swin Transformer, it achieved an increased AUC of 0.019 and an ACC improvement of 1.8%. Against DeiT, our model showed further superiority with a 0.024 increase in AUC and a 2.1% boost in ACC. LeVit and PVT, while efficient in their respective domains, lack in the depth and complexity of feature extraction crucial for medical imaging. Therefore, our model to outperform LeVit with a 0.015 AUC and 1.4% ACC increase, and PVT with a 0.019 AUC and 1.6% ACC improvement.

Consequently, LeVit and PVT struggle with medical image tasks due to their limited ability to capture detailed features and global context. In contrast, Vit-Large’s global self-attention mechanism excels at extracting complex, high-level features essential for accurate PGN and SLA classification in CT images. Its architecture is well-suited for tasks requiring precise feature extraction and detailed analysis.

### 4.5 T-SNE analysis of original image and model-extracted features

**Figure 7:**
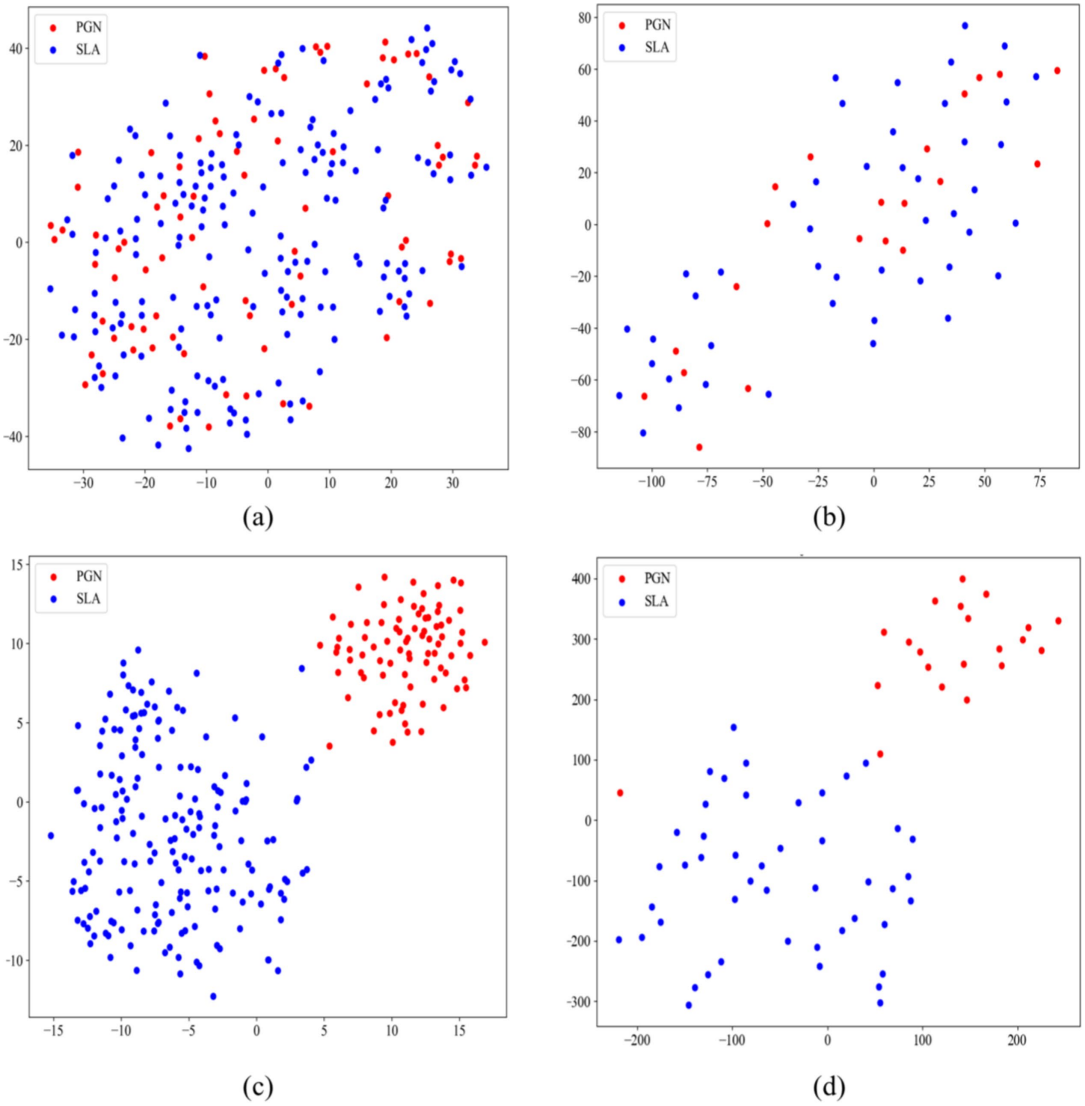
T-SNE projections demonstrating the comparative analysis of original and model-extracted features for PGN and SLA classification. **(A)** The original images of the training set; **(B)** The original images of the testing set; **(C)** The model-extracted features of training set; **(D)** model-extracted features of testing set.

We employed t-SNE for dimensionality reduction on both original images and high-dimensional feature maps extracted by our model from the training and test sets (47). Figure 7A displays the 2D t-SNE projection of the original training set images, where PGN and SLA are intermixed. This mix illustrates that the raw images of PGN and SLA lack distinct, separable features in a lower-dimensional space, highlighting the complexity of classifying these conditions from raw images alone. In contrast, Figure 7C presents the 2D projection of the high-dimensional feature maps extracted by the model. This visualization shows a clear distinction between PGN and SLA, with only a minor overlap. The marked separation achieved in this projection highlights the effectiveness of our model in extracting and transforming raw image data into a format enriched with discernible features. The distinct clustering of PGN and SLA in this space demonstrates the ability of the model to identify and emphasize unique class characteristics, thereby significantly enhancing the accuracy of medical imaging classification.

Furthermore, Figures 7B,D display the t-SNE results for the original images of the test set and the feature maps extracted by the model, respectively. The resemblance of Figures 7A–D indicates a consistency in the performance of the model. This similarity across training and validation sets indicates the robustness and generalizability of our model, affirming its capability to effectively differentiate between PGN and SLA in varied datasets. In a word, this comparison between the original images and the model-processed feature maps via t-SNE projection powerfully demonstrates the value added by our MAEMC-NET in medical image analysis.

### 4.6 Comparison of reconstructed images between MAEMC-NET model and MAE model

**Figure 8:**
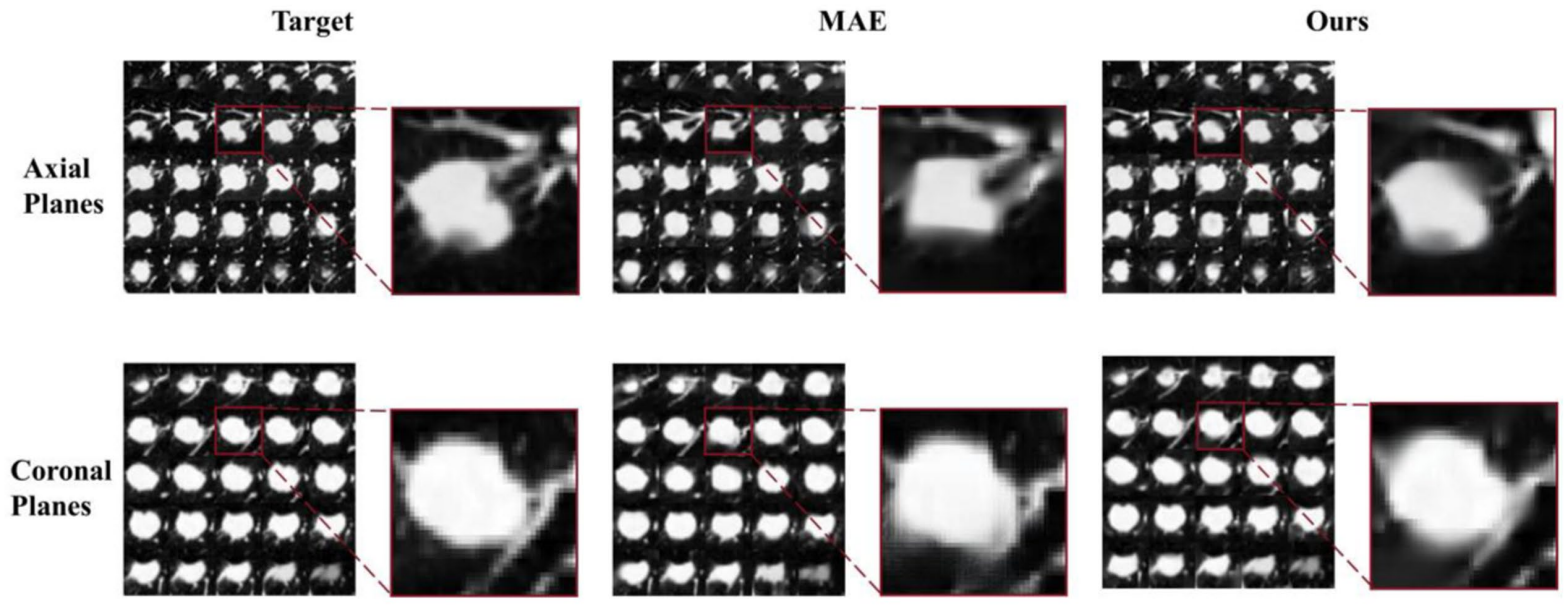
A pair of reconstructed images of axial planes and coronal planes from our model and the MAE model.

To further validate the performance of the MAEMC-NET model proposed in this study, we conducted a comparative analysis by individually assessing the reconstructed images generated by the trained model and those produced by the MAE model against the target images, In Figure 8, we present a series of axial and coronal planes originating from the same lesion, reconstructed separately using the MAEMC-NET model and the MAE model, juxtaposed with the corresponding target images. Each image is accompanied by a magnified view of the same region on its right side, revealing a remarkable resemblance between the reconstructed images of the coronal planes generated by our model and the target images. Furthermore, we computed the Mean Absolute Error (MAE), Mean Squared Error (MSE), Structural Similarity Index (SSIM), and Peak Signal-to-Noise Ratio (PSNR) values for both sets of images, which are summarized in Table 6.

**Table 6:** Result of our models and MAE.

Model	Axial planes				Coronal planes			
	MAE	MSE	PSNR	SSIM	MAE	MSE	PSNR	SSIM
MAE (23)	11.17 ± 2.01	505.02 ± 264.84	21.09 ± 1.94	0.81 ± 0.02	8.92 ± 1.16	327.91 ± 172.24	22.97 ± 1.54	0.86 ± 0.02
Ours	12.03 ± 2.13	628.21 ± 325.71	20.15 ± 1.63	0.78 ± 0.02	8.20 ± 1.74	250.39 ± 157.63	24.14 ± 1.86	0.87 ± 0.02

When the reconstructed images were obtained from the axial planes of the encoder and decoder modules of the training model, the corresponding MAE, MSE, SSIM, and PSNR values were 12.03 ± 2.13, 628.21 ± 325.71, 20.15 ± 1.63, and 0.78 ± 0.02, respectively, slightly lower than those of the MAE model. However, when the reconstructed images were sourced from the coronal planes of the momentum encoder module of the training model, the MAE, MSE, SSIM, and PSNR values were notably superior at 8.20 ± 1.74, 250.39 ± 157.63, 24.14 ± 1.86, and 0.87 ± 0.02, respectively, outperforming those of the MAE model.

Axial slices are effective for localizing small lesions like PGN, providing a clearer cross-sectional view to assess size and relationship with surrounding structures. However, due to their anatomical limitations, they perform slightly worse in overall reconstruction quality compared to coronal slices. Coronal slices offer more comprehensive spatial information, crucial for evaluating tumor invasion and metastasis, especially in SLA staging. Due to the specific design of the MAEMC-NET model, wherein the coronal planes are solely utilized for the contrastive SSL task module and do not directly engage in the training of the generative SSL task module, these planes remain invisible to both the encoder and decoder components used for image reconstruction in both the MAEMC-NET and MAE models. Thus, when the coronal planes are employed for image reconstruction, the resulting MAE, MSE, SSIM, and PSNR values surpass those of the MAE model, thus demonstrating the superior capability of MAEMC-NET in capturing and utilizing information from the coronal planes for enhanced image reconstruction. This underscores the model’s effectiveness in leveraging multi-modal features and exploiting contextual information, leading to improved accuracy and fidelity in medical image reconstruction tasks.

## 5 Conclusion

In conclusion, our study introduces MAEMC-NET, a novel SSL model specifically designed to address the classification of PGN and SLA from CT images. This model successfully amalgamates the strengths of both contrastive and generative SSL techniques. This synthesis enables the model not only to adopt a global perspective in feature extraction from medical images but also to meticulously examine unique image areas, significantly enhancing the generalizability of prototype representations. Our approach represents a groundbreaking SSL method that fully leverages the comprehensive contextual information present in medical imaging. Differing from traditional 2D medical imaging methods, it facilitates the extraction of multifaceted lesion features, ensuring thorough data representation and maximizing the informational content of samples. Additionally, we incorporated a Feature Flattening module, effectively reducing the dimensionality of lesion features extracted by the ViT model. Extensive comparative and ablation studies have confirmed the significant advantages of MAEMC-NET in the classification tasks of PGN and SLA.

Looking ahead, there are several potential avenues for further enhancing the performance of MAEMC-NET. First, integrating other advanced image processing techniques, such as hybrid 3D convolutional networks or attention mechanisms, could improve the model’s ability to capture complex spatial relationships in medical images. Second, the incorporation of more diverse and larger clinical datasets, potentially from multi-center studies, could help to further validate the generalizability of the model and enable it to handle the variations present in clinical environments. Additionally, fine-tuning the model with more advanced semi-supervised or unsupervised learning strategies, such as few-shot learning or active learning, could enhance its performance on limited labeled data, a common challenge in medical image analysis. Finally, future research could explore the integration of MAEMC-NET with real-time clinical decision support systems, enabling seamless adoption in clinical workflows and aiding radiologists in making more accurate diagnoses.

Overall, this research provides an efficient and practical new method for the classification of PGN and SLA, laying a solid foundation for future clinical applications and research.

## Data Availability

The raw data supporting the conclusions of this article will be made available by the authors, without undue reservation.
